# Cloud icing by mineral dust and impacts to aviation safety

**DOI:** 10.1038/s41598-021-85566-y

**Published:** 2021-03-19

**Authors:** Slobodan Nickovic, Bojan Cvetkovic, Slavko Petković, Vassilis Amiridis, Goran Pejanović, Stavros Solomos, Eleni Marinou, Jugoslav Nikolic

**Affiliations:** 1 Republic Hydrometeorological Service of Serbia, 11000 Belgrade, Serbia; 2 National Observatory of Athens, Athens, Greece; 3 Academy of Athens, Athens, Greece; 4 Deutsches Zentrum für Luft- und Raumfahrt (DLR), Institut für Physik der Atmosphäre, Weßling, Germany

**Keywords:** Atmospheric science, Atmospheric dynamics

## Abstract

Ice particles in high-altitude cold clouds can obstruct aircraft functioning. Over the last 20 years, there have been more than 150 recorded cases with engine power-loss and damage caused by tiny cloud ice crystals, which are difficult to detect with aircraft radars*.* Herein, we examine two aircraft accidents for which icing linked to convective weather conditions has been officially reported as the most likely reason for catastrophic consequences. We analyze whether desert mineral dust, known to be very efficient ice nuclei and present along both aircraft routes, could further augment the icing process. Using numerical simulations performed by a coupled atmosphere-dust model with an included parameterization for ice nucleation triggered by dust aerosols, we show that the predicted ice particle number sharply increases at approximate locations and times of accidents where desert dust was brought by convective circulation to the upper troposphere. We propose a new icing parameter which, unlike existing icing indices, for the first time includes in its calculation the predicted dust concentration. This study opens up the opportunity to use integrated atmospheric-dust forecasts as warnings for ice formation enhanced by mineral dust presence.

## Introduction

There have been more than 150 accidents reported by commercial airplanes^1,2^ related to cloud ice impacts, such as engine power loss, blade damage, and the icing of instrument sensors. Most of these incidences have been linked to icing in the upper troposphere in the vicinity of deep summer convection systems. They have often been recorded in tropical and subtropical regions where ice crystals are formed in the absence of supercooled liquid water^2,3^. Cockpit radars generally cannot detect such small ice crystals^4^. High numbers of potentially engine-damaging ice particles typically exist in the outflow of the broad anvils associated with the convective storm complexes^5^. These ice particles can obstruct the normal functioning of important aircraft instruments (e.g., the Pitot tube and air pressure sensors), confuse the aircraft crew and therefore seriously degrade the security of aircraft operations.

## Two catastrophic aircraft accidents caused by icing

In this study, we examine the role of mineral dust on ice formation along the routes of two flights with catastrophic outcomes: the June 2009 Air France flight (AF477) and the July 2014Air Algérie flight (AH5017). To reconstruct the atmospheric and desert dust conditions related to two accidents, we use simulations of the DREAM dust-atmosphere transport model^6,7^ combined with available satellite observations.

Since many aircrafts pass through regions with ice crystals with no fatal consequences, the question that puzzles us herein is what made these two accident flights different from the others. Our analysis shows that for both accident episodes, airborne mineral dust was abundant. As an efficient and dominant ice nucleus in the upper troposphere, dust could enhance the icing process^8^. Another common feature for the flights was that the aircrafts crashed in the vicinity of deep convection systems where ice crystals could not be recorded by aircraft radars, thus leaving pilots unaware of possibly dangerous flight conditions. Finally, the official investigations reported that icing was the cause of both accidents. Namely, the final report for AF477^9^ indicated that '… following the obstruction of the Pitot probes by ice crystals, the speed indications were incorrect and some automatic systems disconnected…'. In the case of AH5017, the final report^10^ stated that '…the airplane speed decreased due to the obstruction of the pressure sensors, probably caused by ice crystals…'.

The AF447 accident happened in the early morning of 1 June 2009 when the airplane crashed into the tropical Atlantic on its way from Rio de Janeiro to Paris^9^. According to the BEA Final Report^9^, the aircraft crashed at approximately 2:00 UTC, at the approximate location of 31° W; 2.7° N and at a height of 11 km. While passing the periphery of a convection zone shortly before the crash, the cockpit speed information abruptly became invalid and confused the crew because all three sensors for the aircraft’s speed (Pitot tubes) were iced. The erroneous information that confused the pilots triggered a series of bad decisions that finally led to the catastrophic accident with 228 human casualties.

**Figure 1:**
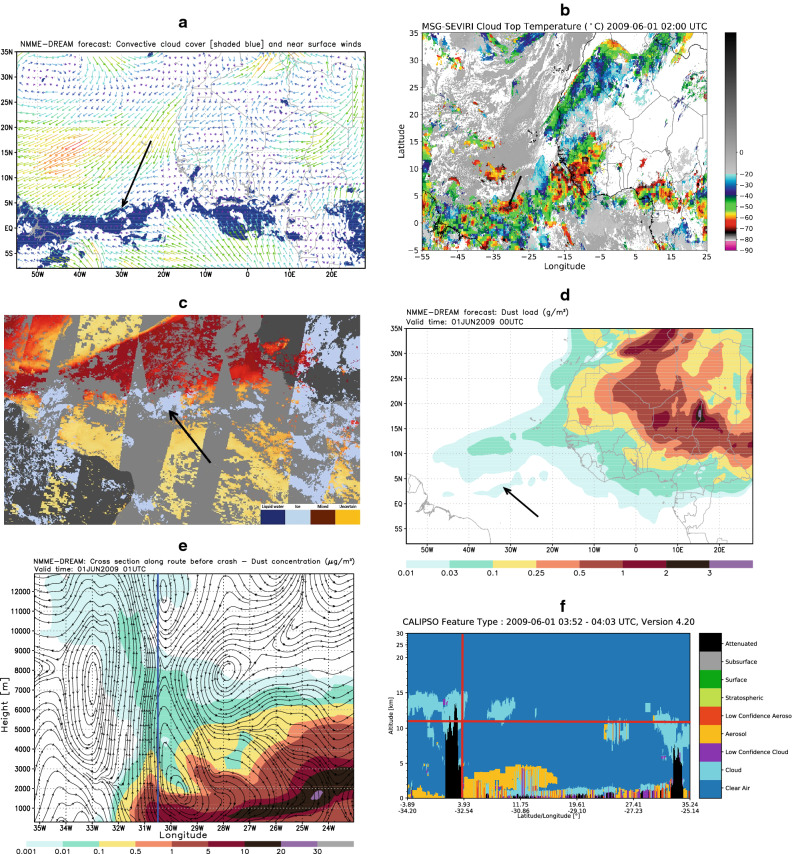
Atmospheric and dust conditions relevant for the AF477 accident. In the above images, arrows and thick lines indicate location of the accident. (**A**) Model convective cloud cover (shaded) and the near-surface wind (colored arrows) at 01 UTC 1 June 2009 indicating the location of the ITCZ characterized with strong wind convergence in the lower atmosphere and by the associated convection. (**B**) Cloud top temperatures observed by EUMETSAT MSG SEVIRI at 01 UTC 1 June 2009 showing the cluster of cumulonimbus clouds at the accident location. (**C**) Dust optical depth (NASA/SUOMI satellite) and ice cloud phase (NASA/MODIS satellite) observed on 31 May 2009 demonstrating the existence of a large-scale dust transport in Central Atlantic combined with the presence of cold clouds in the accident zone. (**D**) Vertically integrated dust concentration [${\upmu {\text{gm}}^{ - 2} }$] at 01 UTC 1 June 2009 as predicted by the model. (**E**) Dust concentration [${\upmu {\text{gm}}^{ - 3} }$] and wind streamlines at 01 UTC 1 June 2009 in the vertical section crossing the atmosphere along the AF477 flight. Dust was lifted by strong upward convection motion from the lower levels to the upper troposphere. The vertical red tick line indicates the accident location (**F**) CALIPSO/CALIOP profile at 02:14 UTC, 1 June 2009 shows cold clouds in the upper troposphere and dust in its lower part.

While crossing the Intertropical Convergence Zone (ITCZ), the aircraft entered a region of strong convection. Figure 1A shows a typical ITCZ pattern on 31 May 2009, as predicted by the atmospheric component of the DREAM model. The pattern is associated with a well-organized convergence of near-surface winds, accompanied with a system of convective clouds. At the same time, cloud-top temperatures observed by EUMETSAT’s MSG SEVIRI instrument indicated the existence of strong convective instability in the zone of the accident (Fig. 1B). In addition, the ice cloud phase observed by NASA/SUOMI NPP satellite coincided with a Saharan dust pattern recorded by NASA/MODIS in the accident region (Fig. 1C). Dust presence in the region was the result of a major desert dust storm developed over Western Africa in the period of 30–31 May and then transported toward the Central Atlantic.

To examine the influence of the airborne dust on the icing process at the time and location of the accident, we performed numerical simulations of mineral dust transport. The model experiment covers the period 24 May–1 June 2009. The initial state of the dust concentration in the model is defined by the 24-h forecast taken from the previous day’s model run except for the ‘‘cold start’’ of the model on 00:00 UTC 24 May 2009, when the initial dust concentration was set to zero. The simulated large-scale pattern dust load (columnar concentration) over the Atlantic (Fig. 1D) is in accordance with the MODIS observation shown in Fig. 1C. The vertical cross section along the AF447 path shows that dust from the lower atmosphere was lifted to the upper troposphere by strong convection and then further laterally spread toward a periphery of convection (Fig. 1E). The model simulates dust concentrations at the order of ~ 0.001 ${\upmu {\text{gm}}^{ - 3} }$ at the level of the flight (~ 11 km). The NASA/CALIPSO (Cloud-Aerosol Lidar and Infrared Pathfinder Satellite Observations) satellite, which crossed the zone of the accident 1 h after the crash, shows the presence of clouds in the layer 10–14 km. NASA/CALIPSO also shows the presence of both ice clouds and desert dust in the wider zone of the accident (Fig. 1F).

**Figure 2:**
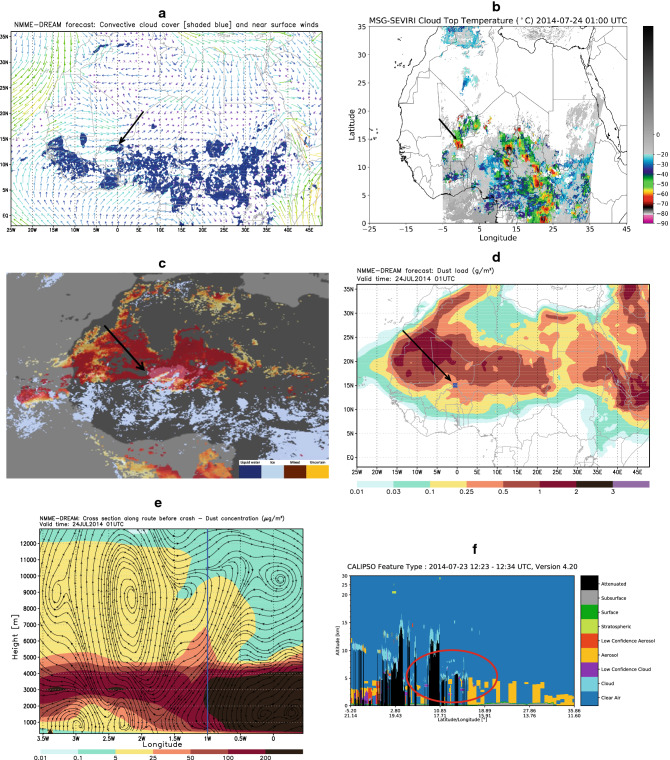
Atmospheric and dust conditions relevant for the AH5017 accident in the Sahel/Sahara region. Arrows and thick lines indicate location of the accident. (**A**) Model convective cloud cover (shaded) and the near-surface wind (colored arrows) at 01 UTC 24 July 2014 indicating the location of WAM characterized with wind convergence in the lower atmosphere. Moisture transported by SE wind generated unstable convection conditions in the region. (**B**) Cloud top temperatures observed by EUMETSAT MSG SEVIRI at 02 UTC 24 July 2014 identifying the presence of cumulonimbus clouds. (**C**) Ice cloud phase in NASA/MODIS satellite observation on 23 July 2014 showing a cold cloud system in the zone of the accident. (**D**) Vertically integrated dust concentration gm^−2^ at 01UTC 24 July 2014 predicted by the model. (**E**) Dust concentration [${\upmu {\text{gm}}^{ - 3} }$] and wind streamlines at 01 UTC 24 July 2014 in the vertical section crossing the atmosphere along the AH5017 flight. Dust was lifted by strong upward convection motion from the lower levels to the upper troposphere. (**F**) CALIPSO/CALIOP profile at 12:15 UTC 23 July 2014, 14 h before the accident (this was the closest time to the accident time in CALIPSO passes over the region) showing that there was already a layer of dust in the lower atmosphere and convective cold clouds at 9–10 km in the wider zone of the AH5017 accident. The red oval tick line shows that the wider region of the accident was characterized by presence of dust, convection and elevated cold clouds.

The AH5017 accident of 24 July 2014 also occurred in the presence of icing conditions. AH5017 was flying from Ouagadougou (Burkina Faso) to Algiers (Algeria). According to the BEA Final Report^10^, the aircraft crashed at approximately 24 July 1:50 UTC, at the approximate location of 1° W; 15° N and at 9.5 km height. The aircraft was crossing the West African Monsoon (WAM) zone, which is a typical system affecting the region during summer. The WAM is characterized by an intertropical front between dry air over the Sahara and moist air from the Atlantic^11^. In the wider region of Africa where the AH5017 accident happened, the model simulation shows a convective precipitation pattern and associated low-level winds (Fig. 2A). Convection was generated by converging air flow in the lower atmosphere. Between 23 and 24 July, the frontal system created thick convective clouds between Burkina Faso and Mali, as depicted by the MSG SEVIRI imagery (Fig. 2B). The air-control authorities advised pilots to change their originally planned flight routes in order to avoid flying through the region with strong convection. The alternative aircraft path crossed the periphery of the convective system. A pattern of ice clouds was observed by NASA/SUOMI on 24 July 2014 in the flight zone, partly overlapping with dust observed by NASA/MODIS (Fig. 2C). Saharan dust was covering a large part of the Sahel and Sahara, as predicted by the model (Fig. 2D). According to the predicted vertical cross section, a part of the dust concentration was lifted and laterally spread by convection from the lower dust layer (Fig. 2E). Twelve hours before the accident, NASA/CALIPSO observed the convective instability was in tandem with the desert dust (Fig. 2F).

According to the official report^10^, ice crystals rather than supercooled water were likely present along the route crossing the periphery of the convective system. The engine pressure ratio, which is the major parameter regulating the engines functioning became erroneous on both aircraft engines because the instrument sensors were obstructed by ice crystals. The pilots did not turn on a de-icing system since they were not warned by the cockpit instruments about the presence of ice. As a result, the aircraft lost its speed, descended and tragically crashed. BEA reported, although with no further elaboration the possible influence of mineral dust, specifically that '… the high dust content was combined with convection over the northern area of the Sahel…' and specifically that '… the concentration of dust may affect the quantity and concentration of condensation nuclei in cumulonimbus.’^10^. Furthermore, BEA stated that '… the analysis of the satellite imagery evolution did not indicate supercooled water was present… The cell was in the "mature stage” when the airplane passed. The anvil cloud was then well developed and the updrafts in the cell core fed this anvil with ice crystals. Thus, the presence of ice crystals within the anvil cloud was very likely'.

## Predicting ice formation caused by mineral dust

The above analysis suggests that there are certain similarities in both accidents. First, a near-surface dust load was lifted by convection and laterally spread along anvils through which both aircraft were passing. Another common feature was that the flights were passing through zones where ice crystal icing was likely dominating over super-cooled water freezing^2^.

Following the needs of aviation for timely and reliable information on expected icing on aircraft cruising routes, several icing indices/parameter shave been developed and routinely implemented in order to identify conditions for icing occurrence^1,12–14^. These methods use either predicted or observed temperature, cloud liquid water, relative humidity and vertical velocity to estimate the icing intensity. Most of them are applied to the mid-troposphere and none considers dust as a contributing factor.

Taking into account the importance of mineral dust in the ice formation process, we here introduce a new icing index, which for the first time considers both thermodynamic and dust parameters. To formulate the new index, we first calculate the number of dust particles participating in ice nucleation n_INP_[#/l], as predicted by the ice nucleation parameterization in the model^7^. The parameterization is based on the immersion nucleation scheme for temperatures between − 15 and − 35 °C and on the deposition nucleation parameterization for temperatures lower than − 35 °C^15^. The derived number of activated dust particles (n_INP_) is a function of predicted temperature, moisture and dust concentration.

**Figure 3:**
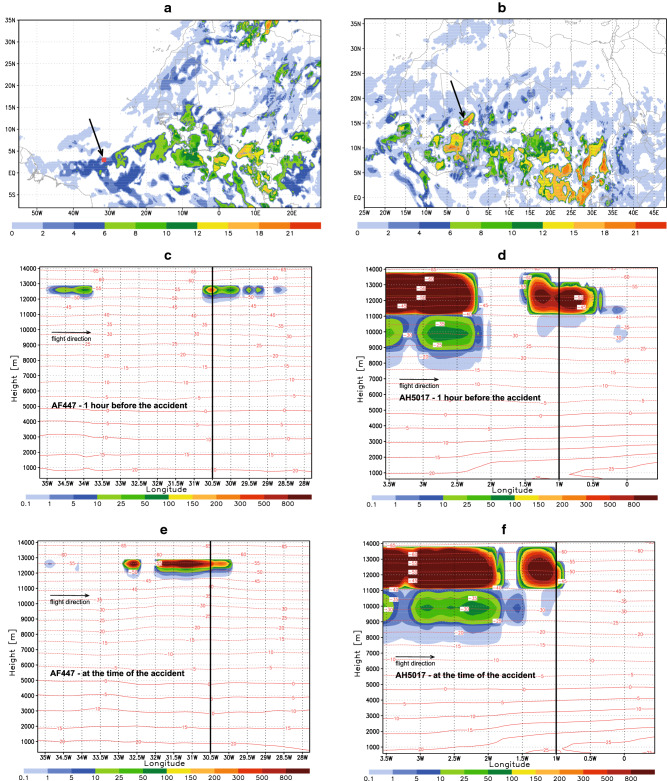

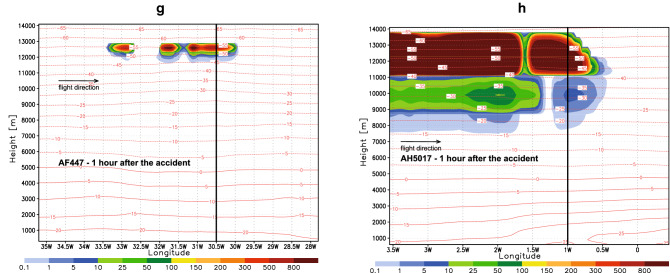
Cloud icing conditions for AF477 (left column) and AH5017 (right column). Arrows, crosses and thick lines above indicate locations of the accident. (**A**, **B**) Predicted vertically integrated log_10_ (*n*_*INP*_) at the times of accidents. (**C**–**H**) Height-time graphs of the predicted Dust Icing Index $DII$ for three consecutive times around the instances of the accidents.

For the considered accidents, we calculate the predicted vertically integrated log_10_(*n*_*INP*_) which indicates the existence of icing conditions in the regions of AF447 and AH5017 flights (Fig. 3A,B). However, such information is too general to be of practical use for flight operations. We therefore propose a parameter that more specifically reflects the icing conditions. Ice nucleation in high cold clouds such as anvil cirrus is affected not only by conventional thermodynamic parameters and aerosol concentration but also by vertical velocities^16,17^. Under conditions prevailing in peripheries of convective systems (e.g., low vertical turbulence in anvils), even lower updraft grid-scale velocities can contribute to ice production. To link the icing intensity with the number of ice nucleating dust particles lifted from lower levels, we define the Dust Icing Index ($DII$) as follows:

$$ DII = n_{INP} w $$

Here, $w$ [hPa/s] is the model vertical velocity. $DII$ represents the vertical flux of $n_{INP}$.

To explore the capability of $DII$ to identify the increased icing, we calculate it along the routes of two considered flight. Figure 3C–H show the $DII$ time evolution as a vertical cross section along the flight paths for three consecutive times around the instances of accidents. For both flights, $DII$ exhibits a sharp rise along a rather narrow vertical layer in the vicinity of the accidents. The index has increased at both accident times and locations. In the case of AH5017 (which occurred at 9.5 km height), $DII$ starts taking significant values within the 9–10 km layer and reaches a maximum above.

**Figure 4:**
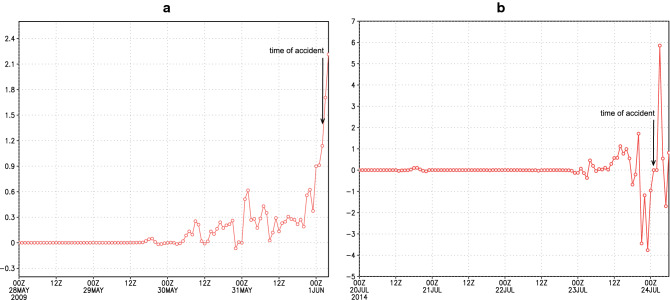
(**A**, **B**) Time evolution of Dust Icing Index $DII$ at the flight levels of AF477 (left) and AH5017 (right).

Figure 4A,B displays $DII$ evolution of at the levels of the two flights starting four days before the accidents. For both flights, $DII$ is almost zero or has low values days before the accidents, but it abruptly increases at approximate times of the accidents.

**Figure 5:**
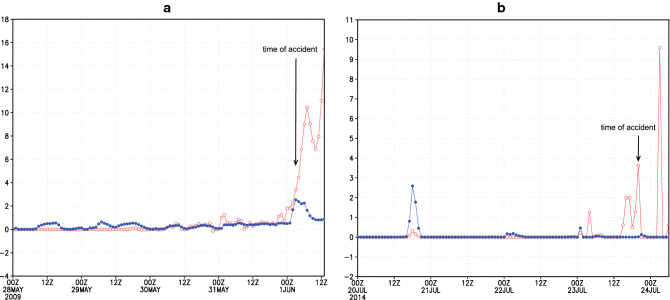
(**A**, **B**) Time evolution of the ice-nucleating dust particle concentration $n_{INP}$ (red) and the ice concentration $n_{ice}$ (in #/liter) of the Ferrier atmospheric driven microphysical scheme^18^ (blue) at flight levels of two considered flights, AF477 (left) and AH5017 (right).

To explore dust relative contribution in forming ice crystals, we compare ice-nucleating dust particle concentration $n_{INP}$ with ice concentration $n_{ice}$ as parameterized by the atmospheric driven microphysical scheme^18^. After reaching the upper-troposphere altitudes at which the accidents happened, it is assumed that at cloud top heights the most of dust particles $n_{INP}$ are activated to ice crystals^19^. Figure 5A,B show that for both considered accidents, dust-caused icing dominates over the icing by other causes.

The successful prediction of risky icing conditions presented herein indicates that the newly proposed Dust Icing Index $DII$ could be a useful warning tool for aviation operators. This study opens an opportunity for detecting flight segments with dangerous icing caused by mineral dust if routine atmospheric-dust forecasts are applied.

## Methods summary

### Modeling

DREAM is an online dust model^6,7,20^ driven by the atmospheric model WRF NMM^21^. There are 28 vertical model levels spanning from the surface to 50 hPa. The horizontal resolution is set to 1/10 deg, for a grid distance of approximately 16 km in the model domain. Here, non-dust ice nucleation process is parameterized by the Ferrier grid-scale cloud microphysics scheme^18^. By numerically solving Euler-based dust mass conservation equations for 8 particle bins with radii spanning from 0.2 to 7.8 µm, DREAM describes all major components of the atmospheric dust processes such as emission, horizontal and vertical turbulent mixing, long-range transport and dust wet and dry deposition. The USGS land cover data combined with preferential dust sources^22^ is used to define barren and arid soils as dust-productive areas. In this study, dust concentration, atmospheric temperature and moisture of the coupled dust-atmospheric model are used to calculate the ice-nucleating dust particles $n_{INP}$. For temperatures in the interval (− 36 °C; − 15 °C), we have implemented the immersion ice nucleation parameterization^23^. For temperatures in the interval (− 55 °C; − 36 °C) under which accidents happened, we use the Ullrich et al., parameterization for the deposition ice nucleation^15^. This scheme is based on the ice nucleation active surface site approach, in which $n_{INP}$ is a function of temperature, humidity and the aerosol surface area concentration.

## Data Availability

For the MSG SEVIRI cloud top temperature plots, we used the product “CLAAS-2.1: CM SAF CLoud property dAtAset using SEVIRI—Edition 2” as described^24^ publicly available from EUMETSAT's Satellite Application Facility on Climate Monitoring (CM SAF) on https://wui.cmsaf.eu/. The CALIPSO data were obtained from the online archive of the ICARE Data and Services center http://www.icare.univ-lille1.fr/archive (NASA/LARC/SD/ASDC. (2018); ICARE Data Center, 2021) NASA/LARC/SD/ASDC. (2018). CALIPSO Lidar Level 2 Aerosol Profile, V4-20 [Data set]. NASA Langley Atmospheric Science Data Center DAAC, retrieved from https://doi.org/10.5067/CALIOP/CALIPSO/LID_L2_05KMAPRO-STANDARD-V4-20, ICARE Data Center: CALIPSO data, available at: http://www.icare.univ-lille1.fr/, last access: 2February 2021.
