# Publisher Correction: Cloud icing by mineral dust and impacts to aviation safety

**DOI:** 10.1038/s41598-021-92428-0

**Published:** 2021-06-18

**Authors:** Slobodan Nickovic, Bojan Cvetkovic, Slavko Petković, Vassilis Amiridis, Goran Pejanović, Stavros Solomos, Eleni Marinou, Jugoslav Nikolic

**Affiliations:** 1 Republic Hydrometeorological Service of Serbia, 11000 Belgrade, Serbia; 2 National Observatory of Athens, Athens, Greece; 3 Academy of Athens, Athens, Greece; 4 Deutsches Zentrum für Luft- und Raumfahrt (DLR), Institut für Physik der Atmosphäre, Weßling, Germany

Correction to: *Scientific Reports* 10.1038/s41598-021-85566-y, published online 19 March 2021

In the original version of this Article, Jugoslav Nikolic was incorrectly listed as a corresponding author. The correct corresponding author for this Article is Slobodan Nickovic. Correspondence and request for materials should be addressed to nickovic@gmail.com.

Additionally, the Acknowledgements section was incomplete.

“Computational resources of the Republic Hydrometeorological Service of Serbia were used to perform the modeling experiments of the study. We acknowledge EUMETSAT and NASA for the use of the satellite data. CALIPSO data were provided by NASA. CALIPSO data were obtained from the ICARE Data Center (http://www.icare.univ-lille1.fr/). We thank the ICARE Data and Services Center for providing access to the data used in this study and their computational center. The MSG SEVIRI data was obtained from EUMETSAT's Satellite Application Facility on Climate Monitoring (CM SAF) (https://wui.cmsaf.eu). The cloud top temperature plots were completed by using data from EUMETSAT’s Satellite Application Facility on Climate Monitoring (CM SAF). The research has been partly supported by the COST Action CA16202 inDust (International Network to Encourage the Use of Monitoring and Forecasting Dust Products) and by the D-TECT Grant (725698) funded by the European Research Council (ERC) under the European Union's Horizon 2020 research and innovation program. EM was funded by a DLR VO-R young investigator group and the Deutscher Akademischer Austauschdienst (Grant No. 57370121).”

now reads:

“Computational resources of the Republic Hydrometeorological Service of Serbia were used to perform the modeling experiments of the study. We acknowledge EUMETSAT and NASA for the use of the satellite data. CALIPSO data were provided by NASA. CALIPSO data were obtained from the ICARE Data Center (http://www.icare.univ-lille1.fr/). We thank the ICARE Data and Services Center for providing access to the data used in this study and their computational center. The MSG SEVIRI data was obtained from EUMETSAT's Satellite Application Facility on Climate Monitoring (CM SAF) (https://wui.cmsaf.eu). The cloud top temperature plots were completed by using data from EUMETSAT’s Satellite Application Facility on Climate Monitoring (CM SAF). The research has been partly supported by the COST Action CA16202 inDust (International Network to Encourage the Use of Monitoring and Forecasting Dust Products) and by the D-TECT Grant (725698) funded by the European Research Council (ERC) under the European Union's Horizon 2020 research and innovation program. EM was funded by a DLR VO-R young investigator group and the Deutscher Akademischer Austauschdienst (Grant No. 57370121). The authors also thank the WMO Sand and Dust Storm Warning Advisory and Assessment System (SDS-WAS) for its support.”

The original Article has been corrected.

